# Search for Proteins in the Liquid Extract of Edible Mushroom, Agaricusbisporus, and Studying their Antibacterial Effects

**Published:** 2012

**Authors:** Mohammad Hassan Houshdar Tehrani, Elham Fakhrehoseini, Mohammad Kamali Nejad, Hadi Mehregan, Mojdeh Hakemi-Vala

**Affiliations:** a Department of Medicinal Chemistry, School of Pharmacy, Shahid Beheshti University of Medeical Sciences, Tehran, Iran.; b School of Pharmacy, Shahid Beheshti University of Medeical Sciences, Tehran, Iran.; c Department of Microbiology, Faculty of Medicine, Shahid Beheshti University of Medeical Sciences, Tehran, Iran.; d Phytochemistry Research Center, Shahid Beheshti University of Medical Sciences.

**Keywords:** Mushroom, *Agaricus bisporus*, Anti bacterial activity, Gel electrophoresis, Protein purification, Chromatography

## Abstract

The edible mushrooms (basidomycetes) have high nutritional value, promote the immune system, and as a source of natural antimicrobial substances have been used to cure bacterial infections since ancient times.Various kinds of proteins with several biological activities are produced by mushrooms. In this research, in order to evaluate antibacterial activity of edible mushrooms, we isolated proteins of *Agaricus bisporus *and examined their effects on gr + and gr- bacteria. Protein extract of the mushroom was first discriminated by homogenation of the chopped fruiting bodies in tris buffer with pH 7.3 and then centrifuged. The Protein concentration was determined by Bradford method. Gel filtration of the proteins was performed by Sephadex G-100 using UV spectrophotometer as detector.Three fractions were collected and their purity level were defined by SDS-PAGE . In order to reach to a more purification level, isolated proteins from the G-100 column were fractionated by the DEAE ion exchange column. Antibacterial activity of total extact proteins as well as protein fractions was evaluated by the method of microdilution against gr+ and gr- bacteria. This study showed that the isolated proteins from the mushroom, *Agaricus bisporus *fruiting bodies were effective against Staphylococcus aureus and MRSA. The proteins of edible mushrooms like *Agaricus bisporus*, maybe viewed as a natural source of antibacterial agents.

## Introduction

Edible mushrooms, macrofungi, have been considered as a source of human healthy food since ancient times (1). In addition, they have been used in traditional medicine to cure various types of diseases (2). Men gain benefit from the natural antibiotics produced by mushrooms to overcome bacterial infections (3). Mushrooms are rich in carbohydrate and protein. Proteins elaborated by these fungi have shown several biological activities like antiproliferative, immunomodulatory, anti viral , antifungal and anti bacterial effects (3-8). There are studies in which the antagonistic activities of some edible mushrooms such as *Agaricus *and *Pleurotus *species on fungi, viruses and bacteria have been demonstrated (9-10). When culture extracts of *Agaricus *sp were assayed *in-vitro *for the antimicrobial effects, inhibitory action on the gr+ and gr- bacterial growth was achieved (9). *E.coli*, *Enterobacter aerogens*, *Klebsiella pneumoniae *were the most sensitive bacteria amongst the tested microorganisms (10) The aim of this study is to throw more light on *Agaricus bisporus *antibacterial action and to find if its activity is reside in its protein content.The result can emphasize the usefulness of the mushrooms as a natural source of antibacterial agents and may open a window to find alternative compounds to substitute the current antibacterial products which are being ineffective by the bacterial resistance. 

## Experimental

All chemicals and culture media were purchased from the Merck and Sigma, Germany. The mushroom, *Agaricus bisporus *provided by a local market was originated from Malard city, central province of Iran. Column beds were purchased from Pharmacia Biotech., Uppsala Sweden. UV spectrometer Model 1501 manufacturer was Shimadzu, Japan. AE-6210 slab gel cast producer was Atto Corporation, Japan. Microplate reader, spectra model was from Tecan, Switzerland. Bacteria with various ATCC numbers were collected from the stock cultures of the Microbiological Lab, Faculty of Pharmacy, Shahid Beheshti University of Medical Sciences, Iran.The bacteria used were: *Staphylococcus aureus*, ATCC 6538; *Bacillus subtilis*, ATCC 6633; *Escherichia coli*, ATCC 8739; *Pseudomonas aeruginosa*, ATCC 9027 and methicillin-resistant *Staphylococcus aureus *(*MRSA*), ATCC 33591.

*Methods*

*Extract preparation*

The fruiting bodies of the mushroom (100 g) prewashed with distilled water were chopped into small pieces and homogenized in their two times volume of cold Tris buffer, 50 mM, pH 7.3 (containing 1.5% PVP , 1% vitamin C, 0.15 M NaCl ) and stirred for 2-3 h. The homogenate was then passed through a cheesecloth and centrifuged (4500 rpm, 30 min).The supernatant was made 70% saturation by ammonium sulphate, or added cold acetone (80% v/v) and left overnight in fridge (4° C). The proteinous precipitate was collected by centrifugation at 4000 × g for 20 min (10 g, wet weight) and stored in freezer until when it is used.

*Column chromatography*

Protein pellet (300 mg) was solubilized in 4 mL of Tris buffer, 0.1M, pH 7.3, and loaded on a Sephadex G-100 column (1.0 × 70 cm) previously equilibrated with Tris buffer. The column was washed with the same buffer and eluates were collected in 3 mL volumes at the flow rate of 40 mL/h and monitored by UV spectrometer at 280 nm.Three protein fractions were obtained as uv absorption dictated, and their purities were checked by SDS-PAGE. The protein content of each fraction was precipitated by adding cold acetone or ammonium sulphate (70 % saturation) and collected after centrifugation (4000 × g for 20 min). Protein fractions thus prepared were then redissolved in Tris buffer and used for the antibacterial activity test. Fraction 2 was further purified by subjecting to an ion-exchange chromatography column of DEAE Sephadex A-50. Elution condition was chosen as a stepwise salt gradient .

*Protein detection and determination*

Sodium dodecyl sulphate-polyacrylamide gel electrophoresis (SDS-PAGE) was performed on the crud protein extract as well as those protein fractions collected from the two chromatography columns, using the method of Laemmli and Favere (11).

Molecular weights of the protein samples were determined by comparison with the molecular mass marker proteins. The protein content of each fraction was calculated by the method of Bradford (12) using BSA as the standard protein.

*Assay of antibacterial activity*

Antibacterial activity of *Agaricus bisporus *protein extract and its fractions was examined against several species of g- and g+ bacteria. The antibacterial test was carried out by the 96-well microplate-based broth dilution method (13). Moller Hilton Broth was used as the liquid culture media for bacteria. Primary bacterial suspension for microplate test was made as 106cfu/mL concentration in MHB (14). The primary protein sample was prepared as 3.2 mg/mL concentration in MBH. 

Preparing the microplate.:100 μL MBH was added to the each well of columns 2-6. 

The wells of columns 1, 2 and 8 were then filled each by 100 μL protein sample. Following mixing, 100 μL protein sample, as 2 times diluted in the wells of column 2 was transferred from column 2 to 3. Again, 100 μL diluted protein sample from column 3 was transferred to column 4 and the process of sample dilution was continued for column 5. From column 5, 100 μL sample was taken and discarded. Now, 100 μL bacterial suspension, prepared as above, was added to the each well of columns 1-6. Column 6 was considered as the bacterial growth control. Column 7 was filled with 200 μL MBH and column 8 was prepared with 200 μL protein sample, in order to be considered as the medium sterility and protein sample sterility controls, respectively. Finally, column 9 was constructed by 100 μL MBH containing Amikacin (32 μg/mL) plus 100 μL bacterial suspension, as a positive antibiotic marker control. It should be mentioned that each row of the microplate prepared as above was allocated to one strain of microorganisms. The microplate was then covered and protected from dehydration with a plastic bag and incubated at 30-35º C for 24-72 h. The amount of microbial growth was measured at time intervals at 570 nm absorbance by the Elisa reader

*Determining IC%*

The percentage of inhibitory concentration of the mushroom protein samples was determined according to the formula as follows;

$\mathrm{IC\%}=\frac{\mathrm{OD}w-(\mathrm{OD}a-\mathrm{OD}c)}{\mathrm{OD}w}$

Where OD_w_ is the absorption of the bacterial growth control, OD_c_ is the absorption of sample containing only protein as a control, and OD_a_ is the absorption of bacterial suspensions containing different amounts of mushroom protein.

## Results and Discussion

The antibacterial activity of, *Agaricus bisporus*, was studied against several species of bacterial pathogens. Table 1 shows the inhibitory effect of an aqueous total protein extract of the mushroom on the growth of some common microbial pathogens. It can be seen that with the reduction of protein concentration from column 1 to 7, bacterial growth increased except for *B. subtilis*. The effect was more pronounced on *S.aureus *and *MRSA *This is demonstrated by % IC determination in Table 2.

**Table 1:** Antibacterial effect of total protein extract of *Agaricus bisporus *on some bacteria ( OD measurement at 570 nm).

	1	2	3	4	5	6	7	8	9
A	0.950	0.995	1.107	1.117	1.130	0.867	0.053	0.382	0.058
B	1.828	1.659	1.648	1.579	1.507	1.462	0.052	0.391	0.059
C	0.590	0.562	0.521	0.450	0.408	0.248	0.052	0.382	0.068
D	0.569	0.507	0.475	0.432	0.410	0.245	0.053	0.389	0.058
E	0.850	0.804	0.743	0.651	0.597	0.604	0.051	0.388	0.059

A: Bacilus subtilis, B: Pseudomonas aeruginosa, C:Staphylococcus aureus,

D: Methicillin-resistant Staphylococcus aureus (MRSA), E: E.coli,

(A-E):1:Protein (1600mcg/mL),2:800 (mcg/mL),3:400 (mcg/mL),4:200 (mcg/mL),

5:100 (mcg/mL), 6: Microorganism, 7: MHB, 8: protein , 9: Amikacin (100 mg/2mL).

**Table 2:** Inhibitory concentration (IC%) of the total protein extract on *S.aureus *and *MRSA*

Concentration(mcg/mL)	1600	800	400	200	100
IC% for *S.aureus*	16٪	27٪	44٪	72٪	90٪
IC% for *MRSA*	26٪	51٪	65٪	82٪	92٪

**Figure 1:**
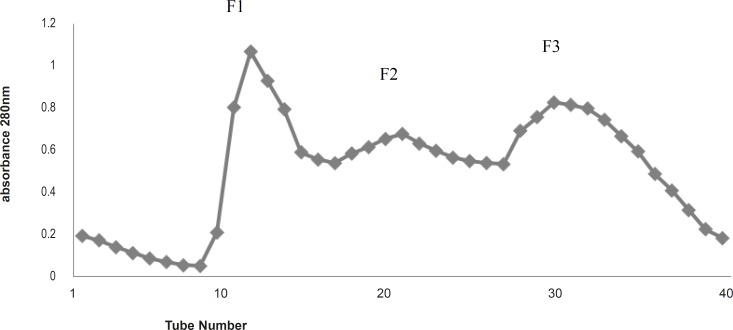
Gel filtration (G-100) Chromatography of the total protein extract of Agricus bisporus

The increased antibacterial activity versus decreased protein concentration may indicate some steric effect of surrounding inert (or inhibitory) proteins covering the active protein(s) ingredient. Gel filtration of the total protein by G-100 column gave three fractions, F_1_-F_3_ ( Figure 1) as UV absorbance at 280 nm indicated. All three fractions were carried out for antibacterial activity experiments. F_1 _and F_3_ did not have a noticeable activity against the bacteria species tested, but F_2_ gave some antibacterial activity (results are not shown).

**Figure 2:**
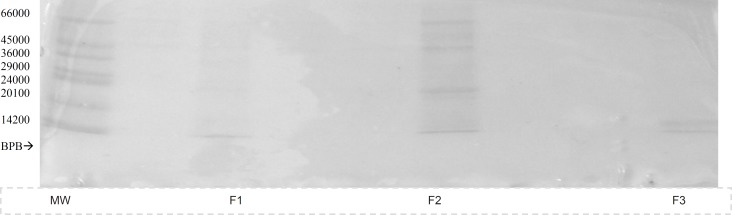
SDS-PAGE of fractions eluted from Gel filtration (G-100) chromatography, MW; molecular mass marker proteins, F1-3 ; protein fractions

The gel electrophoresis, SDS-PAGE, run on all three fractions indicated that F_2_ is the mixture of several proteins (Figure 2), so further purification of F_2 _was carried out using DEAE-A50 ion-exchange column with a stepwise salt gradient elution.

**Figure 3:**
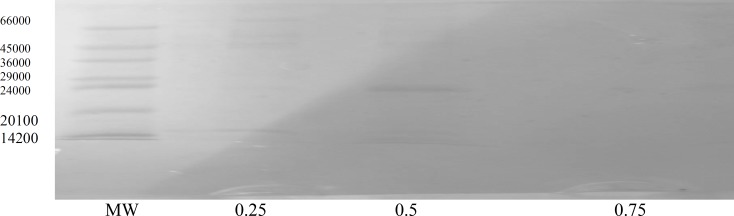
SDS-PAGE of fractions eluted from ion-exchange (DEAE-A50) chromatography with a stepwise salt gradient on F2, MW;molecular mass marker proteins, 0.25, 0.5 and 0.75 show molar salt in Tris buffer

 F_2_ gave almost a pure protein fraction appeared in the tris buffer eluent containing 0.50 salt concentration, as this was demonstrated by SDS-PAGE. (Figure 3).

The molecular weight of this protein was calculated as 22500 Dalton. Antibacterial activity test on the purified F_2_ showed that more than 50% growth inhibition of *S.aureus *as well as *MRSA *can be reached.at the protein concentration of 100 μg/mL. The results are shown in Tables 3-4. These results also infer that the fungal protein has only inhibitory effect on the bacterial species since less protein concentration gives less IC%, *i.e*; the bacterial growth is higher (compare columns 4 and 5 with column 1 in Tables 3 and 4), so the possibility of the fungal protein having any nutrient effect on bacterial growth is rejected. The relative thermostability of the protein may be also inferred by the antibacterial activity test used.

**Table 3:** Antibacterial effect of protein F2 of *Agaricus bisporus *on bacteria (OD measurement at 570 nm)

8	7	6	5	4	3	2	1	
0.160	0.053	0.258	0.279	0.259	0.237	0.211	0.194	A
0.158	0.052	0.281	0.250	0.232	0.210	0.195	0.182	B

A: MRSA, B:Staphylococcus aureus

(A-B):1: Protein (1600mcg/mL), 2: 800 (mcg/mL), 3:400(mcg/mL),4: 200(mcg/mL),5:100

(mcg/mL) ,6: Microorganism ,7: MHB, 8:pprotein,9:Amikacin(100 mg/2mL)

**Table 4:** Inhibitory concentration (IC%) of protein F2 on *S.aureus *and *MRSA*

Concentration(mcg/mL)	1600	800	400	200	100
IC% for *S.aureus*	91%	86%	81%	73%	67%
IC% for *MRSA*	88%	82%	73%	65%	58%

**Figure 4:**
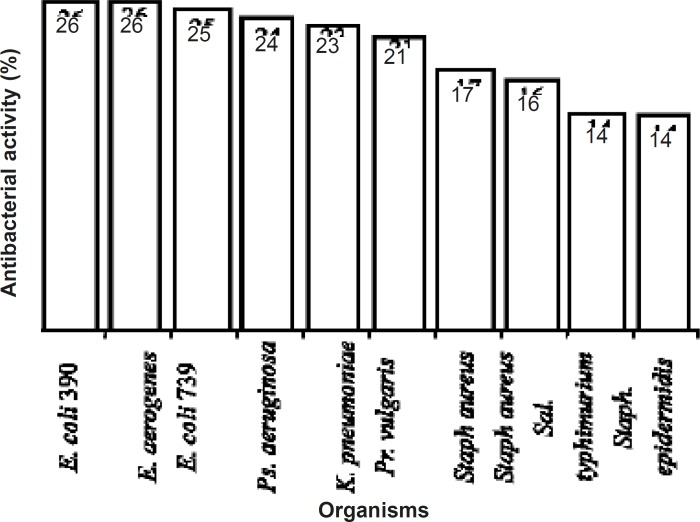
Antibacterial activities of aqueous extracts of *Agaricus bisporus *(10).

The present study showed that *Agaricus bisporus*, an edible mushroom with its enriched protein can have antibacterial properties against some important bacterial pathogens The biological activities of mushrooms have been published by several authors.These properties include antifungal, antiviral and antibacterial activities (6-7,15-18). Mushrooms also have an inhibitory effect on the proliferation of some cancerous cell lines (19,20) The mushroom, *Agaricus bisporus*,has been studied for some biological properties. It was demonstrated that lectin produced by *A.bisporus *has hemagglutinating as well a s antiproliferative activities (19, and refs therein). Some researchers studied on the antibacterial activities of the aqueous and organic extract of the mushroom fruiting bodies (figure 4), but it was not clear that this activity resides in which part of the mushroom ingredients. Also, the concentration of the mushroom aqueous extracts used was not mentioned. This study shows that at least a part of the antibacterial properties of *A.bisporus *belongs to its protein contents. This study also implies that this activity may be important from the point of medical view since two species of important bacterial pathogens were affected by the mushroom protein. The antibacterial activity of this mushroom especially against MRSA may be considered as a good point for searching new antibacterial identities.
